# The route to diagnosis of sarcoma patients: Results from an interview study in the Netherlands and the United Kingdom

**DOI:** 10.1371/journal.pone.0243439

**Published:** 2020-12-07

**Authors:** Vicky L. M. N. Soomers, Winette T. A. van der Graaf, Shane Zaidi, Suzanne E. J. Kaal, Andrew J. Hayes, Bart H. W. B. Schreuder, Robin L. Jones, Ingrid M. E. Desar, Olga Husson

**Affiliations:** 1 Radboudumc, Nijmegen, The Netherlands; 2 Antoni van Leeuwenhoekziekenhuis, Amsterdam, The Netherlands; 3 Royal Marsden Hospital NHS Foundation Trust, London, United Kingdom; 4 Prinses Máxima Centrum, Utrecht, The Netherlands; 5 Institute of Cancer Research, London, United Kingdom; University of Montreal, CANADA

## Abstract

**Introduction:**

Sarcomas are rare tumours. Early diagnosis is challenging, but important for local control and potentially survival and quality of life(QoL). We investigated (1)the route to diagnosis (RtD) experienced by sarcoma patients, including factors contributing to the length of the RtD from patients’ perspective; (2)the impact of the RtD on QoL and care satisfaction; and (3)differences in aims 1–2 between English and Dutch patients.

**Methods:**

Fifteen sarcoma patients from The Royal Marsden Hospital, United Kingdom, and Radboud University Medical Centre, The Netherlands, were interviewed, exploring RtD experiences. Interviews were analysed according to qualitative content analysis.

**Results:**

The main themes were: patient interval, diagnostic interval, reflection on the RtD and recommendations for improvement. Patient interval was long if symptoms were attributed as benign, did not interfere with daily life or were expected to cease. An incorrect working diagnosis, ineffective process of additional investigations, long referral times and lack of a lead clinician lengthened the diagnostic interval. Long waiting times, false reassurance and inadequate information provision led to dissatisfaction and a high emotional burden. Factors for improvement included increasing awareness of patients and healthcare providers, empowering patients, and having a lead clinician.

**Conclusion:**

The RtD of sarcoma patients is complex. Increasing awareness of patients and healthcare providers may contribute to shorten the RtD.

## Introduction

Sarcomas are mesenchymal tumours which comprise more than 70 histological subtypes. They are heterogeneous in terms of age of onset, presentation, anatomic location, speed of progression and clinical outcome. Approximately 85% of sarcomas originate in soft tissue, the remainder in bone. Sarcomas have an estimated incidence averaging 4–5 per 100,000 per year in Europe [1] and are a so-called rare cancer. Patients with rare cancers have a higher mortality rate than those with common cancers because of delays in accurate diagnosis, suboptimal or inadequate treatment, fewer opportunities to participate in clinical trials and less availability of novel agents [2].

**Fig 1:**
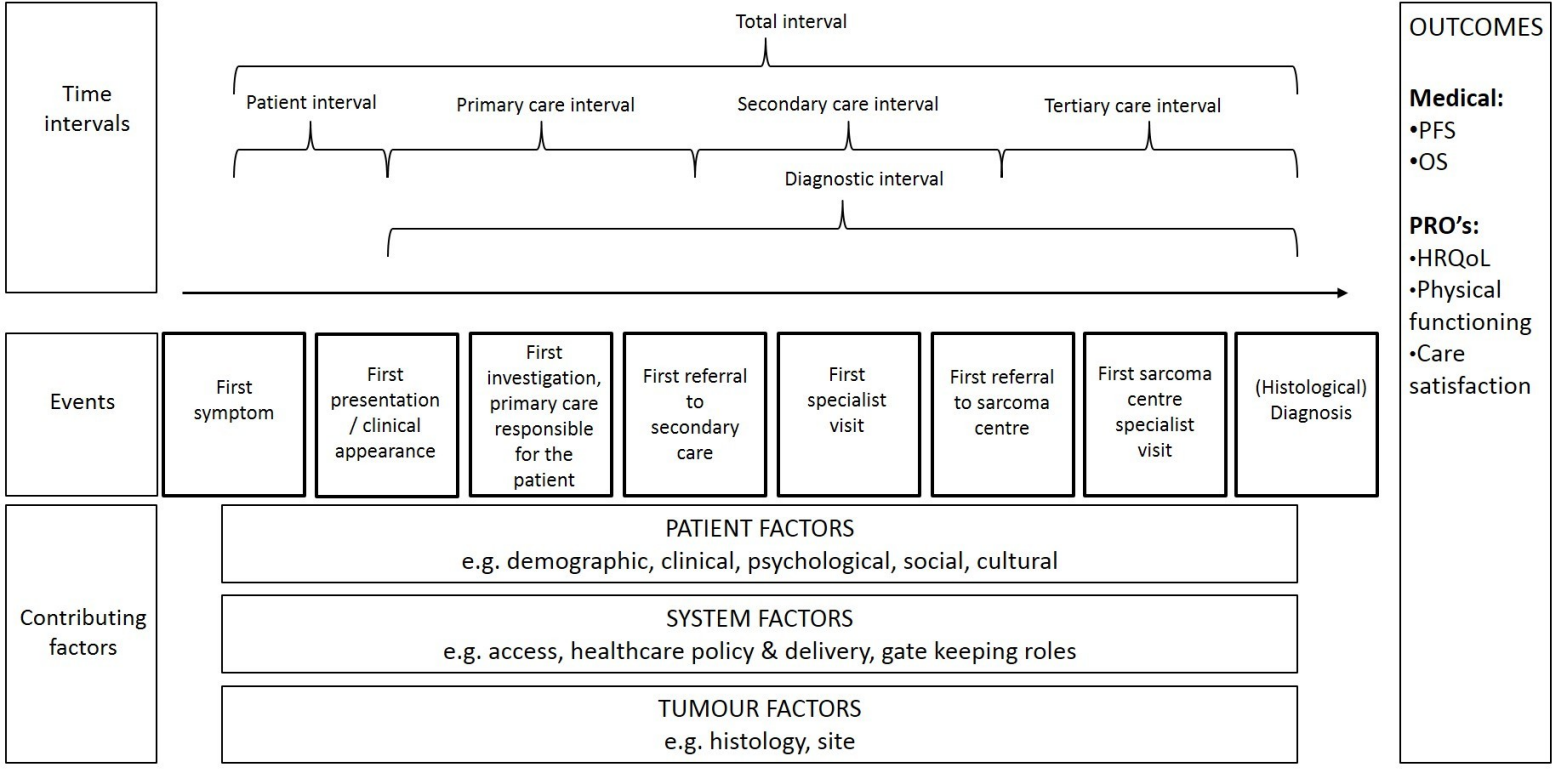
Definitions of the route to diagnosis for sarcoma patients. *This fig was adapted from Olesen et al and Walter et al [9, 13, 14].* >Patient interval: from the date the patient first noticed a sarcoma related symptom until the first presentation to a doctor with this symptom; -Appraisal: from noticing a sarcoma related symptom to deciding to seek the help of a healthcare provider (HCP); -Help-seeking: from making the decision to consult a HCP until the actual appointment; >Diagnostic interval: from first presentation to a doctor until diagnosis; -Primary care interval: from first presentation to a general practitioner (GP) until first referral to secondary (if applicable) or tertiary care, in this study the sarcoma centre (in which referral is the time point at which there is a transfer of responsibility from one HCP to another); -Secondary care interval: from referral to secondary care specialist until referral to tertiary care specialist; -Tertiary care interval: from referral to tertiary care specialist (sarcoma centre) until date of histological diagnosis; >Total interval: from first symptom until histological diagnosis.

Early and adequate diagnosis of sarcoma is challenging due to the heterogeneity in presentation and histology, but is important for local control, and potentially (health-related) quality of life ((HR)QoL) and survival, as seen in other cancer diagnoses [3–8]. Different histological sarcoma subtypes vary in biological behaviour; some aggressive sarcomas cause severe symptoms at an early stage, leading to an early presentation and potentially faster diagnosis, but with a worse outcome than sarcomas that grow slow, causing symptoms with a long total interval. The total interval is the time between first symptoms and (preferably histological) diagnosis (Fig 1) [9].

Apart from cancer characteristics, patient and healthcare factors can be of influence on the length of the total interval [9, 10]. Until now, contradictory results have been found for the influence of patient characteristics [11]; data on comparison of healthcare systems is scarce for sarcomas. The healthcare system of The Netherlands(NL) and United Kingdom(UK) are comparable but differ regarding sarcoma referral pathways.

Patients in both countries have access to health services via a publicly funded healthcare system [12] and generally the general practitioner (GP) is the first healthcare provider(HCP) to be consulted, who refers to secondary or tertiary centres if appropriate. In the UK a minority of patients (11%) is privately insured giving them access to private hospitals via self-referrals, in NL patients may, if they can afford it, use certain diagnostic services provided in private clinics. Sarcoma care has been centralized in the UK, and although bone sarcoma centres exist in NL, care for patients with soft tissue sarcomas has not formally been centralized.

The aim of this study is to investigate (1)the route to diagnosis(RtD) experienced by sarcoma patients, including factors contributing to the length of the total interval from the perspective of a patient; (2)the impact of the RtD length on QoL and care satisfaction; and (3)differences in aims 1–2 between English and Dutch patients.

## Methods

### Conceptual framework

To study the RtD, a framework for research with clear definitions is needed. In this study we combined the widely used models of Olesen *et al* and Walter *et al*, as shown in Fig 1 [9, 13, 14]. The combined model identifies a patient interval, which can be divided between the process of appraisal and help-seeking, and a diagnostic interval, which can be divided into a primary, secondary, and tertiary care interval, the latter was added to fit the sarcoma referral pathway. The treatment interval was left out, as it is out of the scope of this study. The events marking the beginning and end of each interval can be found in Fig 1. The length of each interval can be influenced by patient, system and tumour factors, which will eventually influence clinical and patient-reported outcomes, represented by the outcomes column to the right. This theoretical framework will be used to describe patients’ experience of the sarcoma RtD.

### Study design and patient recruitment

We conducted semi-structured interviews between March and November 2017. Patients were eligible if they were (1)≥18 years; (2)diagnosed within the past 4 months in Radboud University Medical Centre (Radboudumc), NL, or the Royal Marsden NHS Foundation Trust, UK, with histological proven sarcoma and (3)could communicate in Dutch or English, respectively. Patients with significant cognitive impairment or mental health problems, as determined by their HCP, were excluded.

At both sites, eligible patients were identified by their HCP or a member of the research team (VS) and supplied with an information letter. VS then contacted the patients to answer remaining questions and, if agreeing to participate, to set a time for the interview. All participants provided written informed consent before the interviews. Participants completed demographic and clinical questions prior to the interview. RtD, QoL, and care satisfaction were subjects within the interview, and not subject of the questionnaire.

In the UK the study was deemed exempt from full review and approval by a research ethics committee (Committee for Clinical Research, Royal Marsden NHS Foundation Trust), but was approved by the service evaluation committee of the Royal Marsden NHS Foundation Trust (SE669). In NL the study was approved by the medical ethical committee of the Radboudumc (2017–3229).

### Data collection, analysis and reporting

Patients completed a short questionnaire on sociodemographic data and dates of referral. Date of referral to the sarcoma centre and the date of histological diagnosis was confirmed from medical records.

Semi-structured interviews were conducted by one member of the research team at both sites (VS), the interview schedule is provided in S1 Appendix. VS had no clinical relationship with the participants or the treating physician. The interviews were conducted at the hospital. Topics and questions were based on clinical experience and literature review [11, 15–17], whilst emerging questions from interviews were discussed in the following interviews. The interviews were recorded and transcribed verbatim and anonymously. Data analysis was conducted by two coders (VS and OH) using ATLAS.ti 8.0. Data was analysed according to qualitative content analyses and were ordered into relevant code terms, and then categorised into themes by two researchers (VS and OH). Data answering the first research question was organized according to our conceptual framework (directed approach) [18]. The second and third research question were answered by conventional content analyses, in which coding categories are derived directly from the data [18]. After analysing the data independently, the coders discussed and redefined until they reached consensus. Saturation was reached when no new themes were identified. The consolidated criteria for reporting qualitative research (COREQ) were used. S2 Appendix provides an overview of translated quotes.

## Results

### Participants

Seven Dutch and eight English patients with a total interval varying between 10–145 weeks participated. All invited patients gave informed consent and participated in the study. Both patient and diagnostic interval contributed to a prolonged total interval: though the median patient interval was 4 weeks, it ranged from 0–119 weeks; the median diagnostic interval was 18 weeks with a range of 3–140 weeks. More details on these intervals and socio-demographic and clinical characteristics of the patients are presented in Table 1. The study participants reflect the heterogeneity of the sarcoma population.

**Table 1:** Patient and diagnostic interval characteristics.

Patient†	Age	Sex	Diagnosis and site	Total interval (weeks) Median: 24	Patient interval (weeks) Median: 4	Diagnostic interval (weeks) Median: 18	Primary care interval (weeks)	Number of visits / number of doctors seen in primary care	Secondary care interval (weeks)	Number of visits / number of doctors seen in secondary care	Tertiary care interval (weeks)	Number of visits / number of doctors seen in tertiary care
**1**	24	M	High-grade osteosarcoma of the radius	26	3	23	6	2 / 1	13	2 / 1	4	2 / 1
**2**	68	M	High grade peripheral nerve sheath sarcoma of upper leg	49	16	33	1	2 / 1	27	9 / 6	5	1 / 1
**3**	40	F	Solitary fibrous tumour pelvis with multiple metastases	32	0	32	23	2 / 2	7	6 / 4	2	3 / 2
**4**	65	F	Leiomyosarcoma uterus with liver metastases	10	3	7	2	2 / 1	6	6 / 2	0	n.a.
**5**	41	F	High grade osteosarcoma femur	22	11	11	0	1 / 1	1	2 / 1	10	3 / 1
**6**	54	F	Solitary fibrous tumour wrist	137	119	18	4	2 / 1	8	2 / 1	6	2 / 1
**7**	18	M	Ewing sarcoma cerebellum	15	12	3	1	2 / 1	0	1 / 1	2	2 / 1
**8**	69	F	Solitary fibrous tumour groin	13	0	13	5	2 / 1	9	2 / 2	0	n.a.
**9**	85	F	Leiomyosarcoma colon transversum with possible lung lesions	22	9	13	5	2 / 2	9	3 / 1	0	n.a.
**10**	51	F	Low grade endometrial stromal sarcoma uterus	99	57	42	6	2 / 1	36	5 / 1	0	n.a.
**11**	50	M	Extra-osseous Ewing sarcoma thorax	64	4	59	0	1 / 1	56	5 / 3	3	2 / 1
**12**	48	F	Extra -osseous Ewing like sarcoma thorax	145	4	140	137	6 / 2	0	2 / 1	3	3 / 2
**13**	61	M	Retroperitoneal dedifferentiated liposarcoma	24	4	20	14	3 / 3	2	1 / 1	4	2 / 2
**14**	56	F	Undifferentiated pleomorphic sarcoma m. erector spinae	16	1	16	13	3 / 1	0‡	n.a.	3	2 / 2
**15**	69	M	Well differentiated retroperitoneal liposarcoma	10	1	8	0	1 / 1	7	2 / 1	1	2 / 1

†Patients 1–7 were Dutch, patients 8–15 were English. ‡Direct referral from GP to sarcoma centre.

### Themes

**Fig 2:**
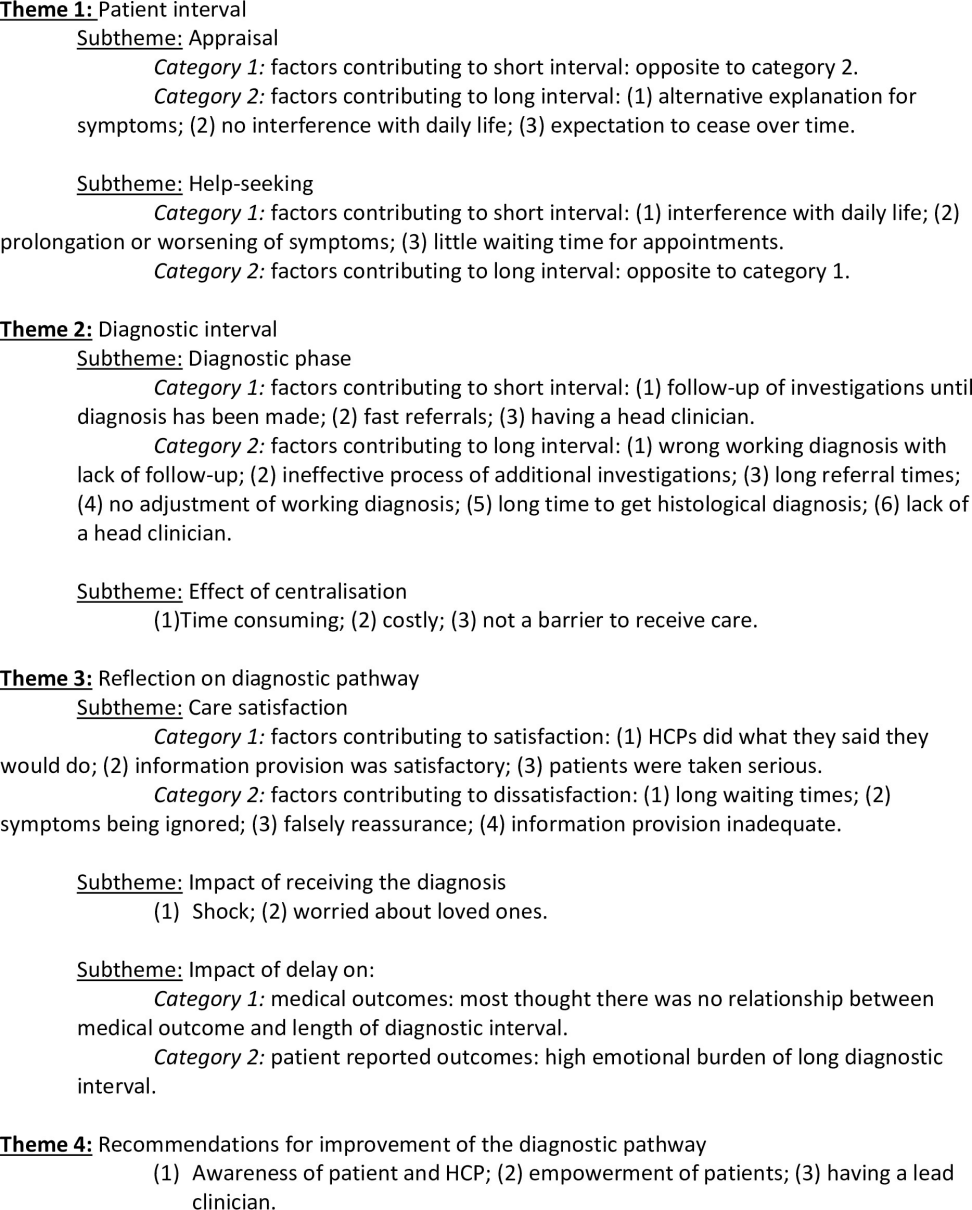
Schematic representation of main findings.

The first research question is answered by the first two themes, which are organized according to our conceptual framework. The other two main themes, ‘reflection of diagnostic pathway’ and ‘recommendations for improvement of the diagnostic pathway’ give an answer to the second aim of this study, to describe the impact of RtD length on QoL and care satisfaction of sarcoma patients. The third research question is accounted for under all themes. Fig 2 shows a schematic representation of our main findings, quotes to support our results can be found in Table 2.

**Table 2:** Quotes.

Theme	Subtheme	Quote
**Patient interval**	Appraisal	*‘Initially we thought it was a*, *like a bug bite’ (male*, *#11)*.
		*‘I stopped doing sport and waited without having any concerns’ (male*, *#1)*.
		*‘I was made this appointment at a back-specialist clinic and again he [a physiotherapist] completely examined me and he quite 100% told me that you have got a class-3 classic sciatica*. *He just said*, *keep taking your medication*, *you’re on the best medication that you can get*. *[*.* *.* *.*] Well*, *with that I then continued’ (male*, *#13)*.
	Help-seeking	*‘I waited until it started to affect my style of life*. *I do a manual job [*.* *.* *.*]*. *So*, *I carried on until basically it started to affect*, *you know*, *my daily work’ (male*, *#13)*.
		*‘Then last year around about Christmas time we noticed it getting much bigger and taking on a red*, *a reddish*, *sort of like a bruise*. *So*, *and it started becoming quite can I say noticeable*. *I couldn’t put my arm down properly and couldn’t sleep on that side*. *And it did start giving me pain’ (male*, *#11)*.
		*‘At that moment the pain became worse*, *[*.* *.* *.*]it really hurt in the buttocks and the back of my legs*. *I thought*, *this really can’t be any good’ (female*, *#4)*.
		*‘What made me decide to [make an appointment]*? *Because it wasn't getting better*. *More frequent I expect and a lot of*.* *.* *.*soreness in the lower abdomen*.*’ (female*, *#9)*.
		*‘I was just getting scared because the pain*, *the pressure in the ribs*, *it wasn’t going away*. *And I did have coughs and things like that*, *then they went away*, *then a lot of mucus then they went away*. *But it just*, *you know*, *something wasn’t right’ (female*, *#12)*.
**Diagnostic interval**	Diagnostic phase	*‘” It’s a hematoma because you’ve obviously pulled a muscle and you know you’ve pulled a muscle and it looks much like a hematoma” they said*. *[*.* *.* *.*] You don’t like to dreadfulize*, *you don’t like to think what ifs*. *I had pulled muscles in the past and they have been painful*, *and I have had problems with back muscles in the past through dangerous sports*, *so I just assumed at the time that they were correct*, *it was a hematoma*.*’ (female*, *#14)*
		*‘Waiting for 6 weeks for an MRI is actually extremely long I think*. *If I would have been aware that it might not be benign*, *I would not have agreed with that*. *But I wasn’t*, *so I just went on a holiday*, *spent the entire day in the sun and drank alcohol*. *I was extremely*, *extremely*, *extremely tired’ (male*, *#1)*.
		*‘The referral for that second opinion at the sarcoma centre had to go through the GP’ (male*, *#2)*.
		*‘The GP never referred me to get a CAT-scan because they [the GP] said I was fit*, *healthy*, *non-smoker*, *never had any family condition or history of anything bad*. *I had no pre-existing conditions of anything and I took no medication at all*, *so they said that there was no reason for a CAT-scan*. *[*.* *.* *.*] So*, *they stopped there and every time I went back they thought it was musculoskeletal’ (female*, *#12)*.
		*‘[*.* *.* *.*] and then a biopsy 3 times*. *They punctured 3 times*, *so they would have enough*, *but they hadn’t because it turned out they were still in doubt between a chondrosarcoma and an osteosarcoma*. *After that I got a biopsy under general anaesthesia and that showed it was an osteosarcoma*, *but low grade*. *[*.* *.* *.*] Then I had an operation*, *which went well*, *but they found other*, *more aggressive*, *cells*, *which is why I had to come here [medical oncology department] eventually’ (male*, *#1)*.
		*‘They had to send the tissue sample to another hospital because they weren't sure*, *I think that's when they pushed back the appointment and then when they had it confirmed at the hospital they brought it [the appointment] forward’ (female*, *#10)*.
		*‘But it wasn’t the same doctor*. *The first time I saw*, *I had seen my own doctor but the second time it was another doctor’ (male*, *#13)*
		*‘So*, *my GP looked and investigated me but did not know what it was*, *so he sent me for an ultrasound that same afternoon’ (female*, *#5)*.
		*‘The doctor said*: *“if I had not seen you on Sunday and I had not seen the difference between Sunday and Friday*, *then I probably would not even have referred you to the hospital”‘ (male*, *#7)*.
	Effect of centralisation	*‘Yes*, *I had to get used to the travel distance*. *[…] The travel costs*, *it was my savings I used*. *You can ask something back from the insurance company*, *that is nice*, *but in the beginning it did cost a bit more money’ (male*, *#1)*.
**Reflection on diagnostic pathway**	Care satisfaction	*‘[Getting the diagnosis took] roughly 4 months*. *If I look back at those dates*, *perhaps two or three weeks might have been saved by a referral being sent on as soon as I was seen as opposed to me phoning up to see if that had gone*. *So*, *apart from that I can't really*, *fault anybody or the process of the system*.*’ (female*, *#8)*.
		*‘I’m very angry*. *I’m not saying that they*.* *.* *. *[pause]*, *it could’ve been done better*. *[*..*] For me to get where I am now I don’t think it would’ve [been necessary]’ (male*, *#13)*.
		*‘Well I suppose if I had been a private patient*, *I would have been seen and all this would have happened maybe half the time or maybe less*. *So on looking at the NHS I'm being realistic and thinking they have done the best they can*.*’ (female*, *#8)*.
		*‘But*.* *.* *.*it's a case*, *when they're all available*. *They are very*, *very pushed*. *I think there's too many patients in our practice anyway*.*’ (female*, *#9)*.
	Impact of receiving the diagnosis	*‘I entered the room and he said*: *“I have not got good news for you”*. *I then thought*: *“wow”*. *It was like a rollercoaster*. *I had gone alone*. *I was startled*, *absolutely*. *No*, *I really did not see it coming*. *It came out of nowhere*.*’ (male*, *#1)*.
		*‘Everybody said the chances of it being anything suspicious were absolutely minute*, *so I had no reason to feel concerned at all*. *To be told*.* *.* *.*actually that's not what you thought it was*. *Alright*, *okay where do we go now*? *[*..*] I was just so surprised after everything being*.* *.* *.*”yes*, *yes everything looks good*, *everything looks good”*. *Then bang*.* *.* *. *[*..*] They don't know how to express too much sympathy*.*’ (female*, *#10)*
	Impact of delay on medical outcomes and quality of life	*‘Yes*, *I understand it does influence my prognosis’ (male*, *#2)*.
		*‘It was difficult from the moment of the result at the hospital until the scans*. *Especially after the scans I was wondering*: *“is it somewhere else*, *how bad is it*?*” I thought I was dying*.*’ (male*, *#1)*.
		*‘I suppose the most difficult part was not knowing*, *or waiting*, *knowing that in all these investigations would obviously help towards the diagnosis and just waiting and not knowing*. *[*..*] But never the less I just got on with life*.*’ (female*, *#8)*.
**Advice Recommendations for improvement of the diagnostic pathway**		*‘Be persistent for what you want [to know]*. *If you don't know [what’s going on] and you want to know then you just have to be persistent about it’ (female*, *#9)*.
		*‘Everything could have gone faster if people had been more aware that this could be sarcoma’ (male*, *#1)*.
		*‘A patient should at the moment of getting complaints of peeing or whatever take it seriously straight away’ (female*, *#3)*.
		*‘The key is to start with your general practitioner’ (female*, *#5)*.
		*‘Possibly my doctor himself should have pushed slightly more rather than saying*: *yes*, *that’s fine*, *you can leave it until after your holidays*, *[the GP] should’ve possibly said*: *“no*, *let’s fix it beforehand”‘ (female*, *#14)*.

### Theme 1: Patient interval: Appraisal and help-seeking

#### Appraisal

Three factors were identified that contributed to a long phase of appraisal. First, many patients had an alternative, benign explanation for their symptoms. In some cases, this alternative explanation was not something a patient would need to seek the help of a HCP for (e.g. a bug bite), while in other cases the symptom was normalised by the patient as being part of life (e.g. old age). Second, if the symptoms did not interfere with daily life, most patients did not feel the urge to seek help. The process of help-seeking was often delayed further due to other life priorities (e.g. a holiday), stopping with activities that became difficult due to symptoms (e.g. gardening), and treatment from a paramedical HCP which decreased complaints temporarily. Third, many patients expected their complaint to cease by itself.

#### Help-seeking

The main trigger of help-seeking was interference of the symptoms with daily life. If symptoms lasted or became worse, this was an extra reason to seek help. There were no delays in making an appointment with an HCP. There were no remarkable differences in the patient interval between NL or the UK.

### Theme 2: Diagnostic interval: Diagnostic phase and effect of centralisation

#### Diagnostic phase

Once the patient seeks help from a HCP, he or she enters the diagnostic phase. This phase was found to be lengthened by six factors, which could send patients back to the process of appraisal.

First, the HCP often had a “working diagnosis” for which wait-and-see management was legitimate. However, a lack of follow-up to ensure resolution of symptoms attributed to a long diagnostic phase.

Second, the process of additional investigations was prolonged: (1) performing investigations was often postponed, (2) passive waiting time for imaging studies and biopsies, (3) long time between investigations and receiving results, and (4) absence of subsequent investigations if results were inconclusive or (false) negative.

Third, referrals took a long time due to: (1) referrals to the wrong specialist, (2) second opinions needed to go through the GP, or (3) long waiting times for an appointment at the sarcoma centre.

Fourth, if the course of disease progression was different than expected, often the working diagnosis was not adjusted, and no new investigations were ordered.

Fifth, if a biopsy was performed it took a long time to get a diagnosis due to the rarity of the disease, heterogeneity and difficulty to diagnose, more tissue needed or necessity to send the material to a sarcoma pathologist.

Sixth, the lack of a lead clinician lengthened the diagnostic process, e.g. when a patient was repeatedly referred to a different specialist at another hospital, or if follow-up consultations did not take place with the same doctor. This was more evident in English than in Dutch interviews, whereas the other causes were mentioned by both groups of patients.

Similarly, the reverse of these factors led to a short diagnostic interval. Especially investigations done within a short time frame, smooth referrals, awareness of the patient that something was wrong and having one lead clinician facilitated fast diagnosis.

#### Effect of centralisation

Many patients acknowledged centralisation of sarcoma care had been difficult in terms of travel distance, arranging transport, the necessity to take longer time off from work, and paying travel expenses from their own savings, especially in the UK. Although travelling to a sarcoma centre or being admitted away from home felt as an effort, it was not a barrier for them to visit a sarcoma centre as they believed they received better care.

### Theme 3: Reflection on diagnostic pathway: Satisfaction with care and impact of delay

#### Care satisfaction

Patients were satisfied about the received care if (1) HCPs kept their promises, (2) information provision was satisfactory, and (3)patients felt they were taken seriously. The absolute length of the diagnostic interval was mostly not of influence on the level of satisfaction with care, but the (subjectively) experienced length was important.

Patients were unsatisfied if (1) there were long waiting times, (2) they felt their symptoms were ignored, (3) they were falsely reassured, and (4) information provision was inadequate. In the UK patients felt that their doctors were busy, and the system was pushed, therefore not faulting their doctor but the system they needed to work in. They thought this caused GP’s to be less proactive and thought waiting times were longer due to the National Health Service (NHS) and would have been shorter if they were privately insured. This was not applicable to Dutch patients.

#### Impact of receiving the diagnosis

Most patients who experienced a long diagnostic interval felt shocked when they received the diagnosis. Those who experienced a short diagnostic interval were more concerned about their loved ones than themselves.

#### Impact of delay on medical outcomes and quality of life

Only one patient thought a long diagnostic interval influenced his clinical outcomes. However, the emotional burden with feelings of anxiety, fear of dying, and anger was mentioned by most patients. The impact of a long interval on quality of life was largest when patients had not expected a malignant diagnosis. They also experienced the period in which they knew they had a malignancy but had to wait for the definite diagnosis as difficult.

### Theme 4: Recommendations for improvement of the diagnostic pathway

Patients gave advice regarding improvements of the diagnostic pathway. First, awareness of both the patient and HCP was described as a requisite for an efficient diagnostic pathway. Awareness leads to investigations being done in a shorter time frame due to persistence of patients or recognized necessity for investigations by the HCP. Second, patients indicated they can be empowered by receiving copies of referral letters. Third, each patient should have a lead clinician, who tracks results of investigations until a diagnosis has been made. Many patients emphasized the importance of the role of the gatekeeper, most often the GP. If the gatekeeper ordered the right investigations or referred them to the correct specialist most RtD’s went smoothly. If the gatekeeper reassured them, referred to the wrong specialist, or postponed investigations or referrals, then patients lingered for a long time. These recommendations were the same among Dutch and English patients.

## Discussion

This study investigated the RtD as experienced by sarcoma patients, its effect on QoL and care satisfaction and differences between English and Dutch patients. We found the RtD to be variable and many sarcoma patients encounter difficulties during the process. The total interval of our patients varied from 10 to 145 weeks, this large variability is concurrent with the literature [13].

Our study identified four main themes: patient interval, diagnostic interval, reflection on diagnostic pathway and recommendations for improvement of the diagnostic pathway; Fig 2 shows a schematic summary of our main findings.

Factors influencing the length of the patient interval were alternative explanations for symptoms, interference with daily life, expectations about prolongation of symptoms and waiting time for appointments. The diagnostic interval was lengthened if the working diagnosis was inaccurate or not adjusted, the process of investigations and receiving their results was inefficient, there was a lack of a head clinician, or there were long referral times. These associations are in concurrence with existent cancer literature, which has shown that key concepts for length of patient and diagnostic interval were recognition and interpretation of symptoms, the impact on everyday life by symptoms, experiences of generalist health care, entry into secondary care, repeated cycles of healthcare seeking and appraisal without resolution and lack of follow-up of persisting symptoms [15–17, 19, 20]. As shown in our study, the role of the GP or subsequent lead clinician is important for referral times and promptness of successive additional investigations.

Sarcoma care is centralized in sarcoma expert centres in many countries, it is generally accepted that this improves diagnosis and subsequent treatment [1, 2]. We explored the effect of centralisation on patients’ experiences of the diagnostic pathway. For nearly all included patients, travelling to a sarcoma centre was time consuming and costly, but not withstanding them from going there to receive the best possible care.

Although the diagnostic interval was perceived more negative by English patients, care satisfaction was equal amongst Dutch and English participants. An interesting finding is that the absolute duration of the diagnostic interval was not of influence on care satisfaction as reported by our participants. They were unsatisfied with passive waiting time, being ignored or falsely reassured, and if information provision was inadequate. The reverse of these factors improved their level of care satisfaction. To the best of our knowledge, the influence of RtD on care satisfaction has not been measured. However, there is a lot of literature and policy reports measuring RtD length including anecdotal evidence that the large number of prolonged time to diagnosis will lead to lower care satisfaction [11, 21, 22]. The European patient advocacy group (SPAEN) has written a position paper in which the first priority challenge is earlier accurate diagnosis [23]. This underlines the importance of a fast RtD for patients. Delays to diagnosis have also been shown to be the main cause of sarcoma litigation [24, 25]. General cancer literature has shown contradictory results about the relation between care satisfaction and experienced RtD; a qualitative study with 26 patients with anal cancer showed most patients were satisfied with received care, unless they experienced passive waiting time [26]. However, another study in 353 women with gynaecological cancer reported a longer diagnostic interval led to lower care satisfaction [7], and a study in 904 cancer patients showed patients had less confidence in GPs after being diagnosed with a doctor delay [27]. Upon receiving the diagnosis patients with a long interval felt shocked, those with a shorter interval were mainly worried about their family.

Research about the effect of the length of total on clinical and patient-reported outcomes is mostly retrospective and shows contradictory results [11]. However, a long total interval could cause significant psychological morbidity [6]. Participants in our study confirm that waiting time causes psychological distress. Yet, only one patient considered his delayed diagnosis to influence his clinical outcome. This is an additional rationale to investigate the effect of the length of diagnostic interval on both clinical as well as patient-reported outcomes.

The fourth theme, recommendation for improvement of the diagnostic pathway, provided us with information consistent with existing cancer literature but now from a patients’ perspective: the main recommendations were to increase awareness amongst patients and HCPs, to empower patients, and to have one lead clinician [10, 15, 20].

This study has given us insight in the patients’ perspective of the diagnostic pathway. Nevertheless, several limitations should be considered. First, although qualitative study methods are ideal to gain insight in what is important and why, this is a descriptive study and results can therefore not be generalized to all sarcoma patients. The RtD is influenced by healthcare systems, culture and organization of care and therefore these results cannot be generalized to sarcoma patients outside NL or the UK. In our analyses, the same factors facilitating and prolonging time to diagnosis emerged from all interviews. In that respect, we concluded we reached data saturation and did not add any more participants. However, due to the heterogeneity of the disease, depending on subtype and location, patients will encounter many different specialist in the RtD. As shown in this study, this results in many different pathways and we cannot say we explored all possible cases. Second, one of the aims of our study was to examine the effect of the RtD on QoL. However, the impact on QoL was not a main issue in our interviews. To investigate whether QoL is truly not impacted by length of the total interval, quantitative research is needed. Third, we left the treatment interval out of the scope of our study. Outcomes may be influenced by this time interval, and some patients had just heard their diagnosis whereas others had already started treatment, which may have influenced their responses. Fourth, patient interval dates were patient-reported and therefore possibly inaccurate. To limit recall bias, we included and interviewed patients within four months after diagnosis, in our clinical experience patients remember many details about their RtD, we therefore think this bias is limited.

Until now, the conceptual framework used to describe the RtD was a theoretical model representing methodological recommendations. Our study described the patient experience of sarcoma diagnosis and for the first time confirms that this patient experience fits the model. This is an important finding, because understanding the RtD from both the patient and clinical perspective is necessary to improve the diagnostic pathway.

Our study confirms the research model as presented in Fig 1 is useful for sarcoma patients and can be used in future research to report uniformly on the subject. The fig could be given more detail specifically for sarcoma patients, e.g. contributing events could be complemented with patient awareness and empowerment for patient factors, direct access to investigations and having a lead clinician are important system factors and care satisfaction is influenced by passive waiting times. However, to make these findings more robust and to study the effect of total interval on outcomes, more research is needed. This qualitative study was used to identify factors important to patients, to use in our design of a large quantitative and prospective longitudinal study, currently a trial in progress (the ‘QUEST’ study, NCT03441906). This study will allow us to quantify total interval and its components, provide insight in factors contributing to its length, and study the relationship between its length and clinical as well as patient-reported outcomes. If the sample is large enough, we may be able to distinguish these features for specific sarcoma subtypes. Hopefully it will allow us to give a more detailed insight in what is specific in the RtD for sarcoma patients, compared to other malignancies. The present study has contributed to understanding patients’ experiences during the diagnostic process and has enabled us to design a study in which the patients’ perspectives are involved.

## Conclusion

This study confirmed RtD for sarcoma patients is variable and found several patient and system factors influencing its length for English and Dutch patients, and described its effect on care satisfaction. The total interval could be reduced by increasing awareness amongst patients and HCPs, having an efficient pathway for investigations and referrals and working with a lead clinician. Centralisation of care is costly and time consuming, but not a barrier for receiving care. The patients’ experience of the RtD could be improved by reducing passive waiting time and providing adequate information. Quantitative research is needed to confirm these findings and study the impact of RtD on clinical and patient-reported outcomes.

## Supporting information

S1 Appendix Interview schedules. (DOCX)

S2 Appendix Translated quotes. (DOCX)

## Abbreviations

RtD: route to diagnosis
HRQoL: health-related quality of life
QoL: quality of life
NL: The Netherlands
UK: United Kingdom
GP: general practitioner
HCP: healthcare provider

## References

[1] Stiller CA, Trama A, Serraino D et al Descriptive epidemiology of sarcomas in Europe: report from the RARECARE project. Eur J Cancer 2013; 49: 684–695. 10.1016/j.ejca.2012.09.011 23079473

[2] Blay JY, Coindre JM, Ducimetiere F, Ray-Coquard I. The value of research collaborations and consortia in rare cancers. Lancet Oncol 2016; 17: e62–e69. 10.1016/S1470-2045(15)00388-5 26868355

[3] DeSantis CE, Kramer JL, Jemal A. The burden of rare cancers in the United States. CA Cancer J Clin 2017; 67: 261–272. 10.3322/caac.21400 28542893

[4] McPhail S, Johnson S, Greenberg D et al Stage at diagnosis and early mortality from cancer in England. Br J Cancer 2015; 112 Suppl 1: S108–115. 10.1038/bjc.2015.49 25734389 PMC4385983

[5] Fern LA, Birch R, Whelan J et al Why can't we improve the timeliness of cancer diagnosis in children, teenagers, and young adults? Bmj 2013; 347: f6493 10.1136/bmj.f6493 24170036

[6] Risberg T, Sorbye SW, Norum J, Wist EA. Diagnostic delay causes more psychological distress in female than in male cancer patients. Anticancer Res 1996; 16: 995–999. 8687166

[7] Robinson KM, Christensen KB, Ottesen B, Krasnik A. Diagnostic delay, quality of life and patient satisfaction among women diagnosed with endometrial or ovarian cancer: a nationwide Danish study. Qual Life Res 2012; 21: 1519–1525. 10.1007/s11136-011-0077-3 22138966

[8] Walming S, Block M, Bock D, Angenete E. Timely access to care in the treatment of rectal cancer and the effect on quality of life. Colorectal Dis 2018; 20: 126–133. 10.1111/codi.13836 28777877

[9] Weller D, Vedsted P, Rubin G et al The Aarhus statement: improving design and reporting of studies on early cancer diagnosis. Br J Cancer 2012; 106: 1262–1267. 10.1038/bjc.2012.68 22415239 PMC3314787

[10] Brousselle A, Breton M, Benhadj L et al Explaining time elapsed prior to cancer diagnosis: patients' perspectives. BMC Health Serv Res 2017; 17: 448 10.1186/s12913-017-2390-1 28659143 PMC5490154

[11] Soomers VLMN, Husson O, Young RJ et al The sarcoma diagnostic interval: a systematic review on length, contributing factors and patient outcomes. ESMO open 2020; 10.1136/esmoopen-2019-000592 32079621 PMC7046415

[12] Mossialos E. WM, Osborn R., Sarnak D. International profiles of health care systems. The commonwalth Fund 2016; Januart 2016.

[13] Olesen F, Hansen RP, Vedsted P. Delay in diagnosis: the experience in Denmark. Br J Cancer 2009; 101 Suppl 2: S5–8. 10.1038/sj.bjc.6605383 19956163 PMC2790711

[14] Walter F, Webster A, Scott S, Emery J. The Andersen Model of Total Patient Delay: a systematic review of its application in cancer diagnosis. J Health Serv Res Policy 2011; 17: 110–118. 10.1258/jhsrp.2011.010113 22008712 PMC3336942

[15] Smith LK, Pope C, Botha JL. Patients' help-seeking experiences and delay in cancer presentation: a qualitative synthesis. Lancet 2005; 366: 825–831. 10.1016/S0140-6736(05)67030-4 16139657

[16] Black G, Sheringham J, Spencer-Hughes V et al Patients' Experiences of Cancer Diagnosis as a Result of an Emergency Presentation: A Qualitative Study. PLoS One 2015; 10: e0135027 10.1371/journal.pone.0135027 26252203 PMC4529308

[17] Gibson F, Pearce S, Eden T et al Young people describe their prediagnosis cancer experience. Psychooncology 2013; 22: 2585–2592. 10.1002/pon.3325 23784978

[18] Hsieh HF, Shannon SE. Three approaches to qualitative content analysis. Qual Health Res 2005; 15: 1277–1288. 10.1177/1049732305276687 16204405

[19] Evans J, Ziebland S, McPherson A. Minimizing delays in ovarian cancer diagnosis: an expansion of Andersen's model of 'total patient delay'. Fam Pract 2007; 24: 48–55. 10.1093/fampra/cml063 17158183

[20] Molassiotis A, Wilson B, Brunton L, Chandler C. Mapping patients' experiences from initial change in health to cancer diagnosis: a qualitative exploration of patient and system factors mediating this process. Eur J Cancer Care (Engl) 2010; 19: 98–109. 10.1111/j.1365-2354.2008.01020.x 19552730

[21] UK S. Delays cost lives, a call to policy makers to improve early diagnosis of sarcoma. Sarcoma UK 2020; sarcoma.org.uk/policy; accessed September 2020.

[22] Neal RD, Tharmanathan P, France B et al Is increased time to diagnosis and treatment in symptomatic cancer associated with poorer outcomes? Systematic review. Br J Cancer 2015; 112 Suppl 1: S92–107. 10.1038/bjc.2015.48 25734382 PMC4385982

[23] Wilson R. The challenge of sarcomas: the patient advocacy group perspective. Sarcoma Patients EuroNet 2019; https://www.sarcoma-patients.eu/en/docman/position-papers/189-the-challenge-of-sarcomas-the-patient-advocacy-group-perspective/file; accessed September 2020. 10.1186/s13569-019-0121-6 31346406 PMC6636022

[24] Harrison WD, Sargazi N, Yin Q, Chandrasekar CR. Delayed diagnosis in primary care-The main cause of sarcoma litigation in the United Kingdom. J Surg Oncol 2016; 113: 361–363. 10.1002/jso.24149 26728703

[25] Mesko NW, Mesko JL, Gaffney LM et al Medical malpractice and sarcoma care—a thirty-three year review of case resolutions, inciting factors, and at risk physician specialties surrounding a rare diagnosis. J Surg Oncol 2014; 110: 919–929. 10.1002/jso.23770 25155556

[26] Chiu S, Joseph K, Ghosh S et al Reasons for delays in diagnosis of anal cancer and the effect on patient satisfaction. Can Fam Physician 2015; 61: e509–516. 26889506 PMC4642922

[27] Larsen MB, Hansen RP, Olesen F, Vedsted P. Patients' confidence in their GP before and after being diagnosed with cancer. Br J Gen Pract 2011; 61: e215–222. 10.3399/bjgp11X572409 21619745 PMC3080226

