# Supplementary material for: The route to diagnosis of sarcoma patients: Results from an interview study in the Netherlands and the United Kingdom
Source: PLoS One. 2020 Dec 7;15(12):e0243439. doi: 10.1371/journal.pone.0243439 (PMC7721153; doi:10.1371/journal.pone.0243439)
Supplement: S1 Appendix — (DOCX) [file pone.0243439.s001.docx]

**Appendix 1: interview schedule**

**Introductory question**

Can you briefly give me the outlines of what happened when you experienced your first symptoms until the moment the diagnosis of sarcoma was made?

**Core questions**

If you think back at the period in which you first noticed your symptoms, what was your explanation for having those symptoms?

If appropriate: What do you think delayed you going to a doctor?

What made you go to the doctor (in the end), and what happened when you did?

During the diagnostic pathway, did finance or travel distance, including taking days off work etc, play a role in the decisions you made? How?

How did you feel about the diagnosis itself?

Looking back at the entire pathway, which period do you feel was the most difficult in a psychological way? Why?

What do you think the effect of the length of the diagnostic pathway is / has been?

Looking back, would you have done anything differently?

Are you satisfied with the care you have received? Why (not)?

**Closing question**

Have you got recommendations on how to improve the diagnostic pathway?
