# Supplementary material for: The route to diagnosis of sarcoma patients: Results from an interview study in the Netherlands and the United Kingdom
Source: PLoS One. 2020 Dec 7;15(12):e0243439. doi: 10.1371/journal.pone.0243439 (PMC7721153; doi:10.1371/journal.pone.0243439)
Supplement: S2 Appendix — (DOCX) [file pone.0243439.s002.docx]

**Appendix 2: translated quotes**

| **Translated quote** | **Original quote** |
| --- | --- |
| ‘I actually had some problems with peeing for some time. You just put it aside as being: ‘you get older, so you may need to go to the bathroom more quickly or just drink more’ (female, #3). | Eigenlijk had ik bepaalde klachten met plassen al langer. Alleen dat zet je dan weg als zijnde: je wordt ook ouder, dus misschien moet je sneller naar de wc of je drinkt gewoon wat meer. |
| ‘I stopped doing sport and waited without having any concerns’ (male, #1). | Ik ben gestopt met sporten en gewoon even afgewacht zonder enige onrust. |
| ‘Waiting for 6 weeks for an MRI is actually extremely long I think. If I would have been aware that it might not be benign, I would not have agreed with that. But I wasn’t, so I just went on a holiday.’ (male, #1). | 6 weken wachten op een MRI is ook eigenlijk extreem lang vind ik. Als ik mezelf er al van bewust was dat het niet goed zou kunnen zijn, dan was ik daar niet akkoord mee gegaan. Dat was ik dus niet, ik ben gewoon op vakantie geweest. |
| ‘The referral for that second opinion at the sarcoma centre had to go through the GP’ (male, #2). | Die verwijzing voor die second opinion hier naar het Radboud moest via de huisarts. |
| ‘They biopsied 3 times so they would have enough, but they hadn’t because it turned out they were still in doubt between a chondrosarcoma and an osteosarcoma. After that I got a biopsy under general anaesthesia and that showed it was an osteosarcoma, but low grade. [...] Then I had an operation, which went well, but they found other, more aggressive, cells, which is why I had to come here eventually’ (male, #1). | ‘Ze deden dat [biopteren] extra 3 keer want dan zouden ze genoeg hebben, maar ook dat was niet genoeg, omdat ze toen aan het twijfelen waren tussen een chondrosarcoom en een osteosarcoom. Daarna kreeg ik een biopt onder narcose en die liet zien dat het een osteosarcoom was, maar laaggradig. […]De operatie gedaan, dat was wel gelukkig goed gegaan, maar dan toch andere agressieve cellen gevonden, waardoor ik toch hier in het traject ben beland. |
| ‘That MRI-centre is nearby and I just went there and said: “I want you to make an MRI of my leg”. They do that if you pay for it yourself’ (male, #2). | Dat MRI-centrum zit in Elst en daar ben ik gewoon langsgegaan en gezegd: 'ik wil dat er een MRI van mijn been gemaakt wordt'. Dat kan als je het zelf betaalt. |
| ‘So, my GP looked and investigated me but did not know what it was, so he sent me for an ultrasound that same afternoon’ (female, #5). | Dus hij heeft gekeken en onderzocht en vond het eigenlijk ook maar, ja, kon het ook niet thuis brengen, dus hij heeft me eigenlijk gelijk ’s middags voor een echo gestuurd |
| ‘The doctor said: “if I had not seen you on Sunday and I had not seen the difference between Sunday and Friday, then I probably would not even have referred you to the hospital”’ (male, #7). | De dokter heeft ook zelf gezegd van: had ik jou die zondag niet gezien en het verschil niet gezien tussen zondag en vrijdag, dan had ik je waarschijnlijk vrijdag niet eens doorgestuurd naar het ziekenhuis. |
| ‘Yes, I had to get used to the travel distance. […] The travel costs, it was my savings I used. You can ask something back from the insurance company, that is nice, but in the beginning it did cost a bit more money’ (male, #1). | Ja, die reisafstand was in het begin wel even wennen. […]Het is een buffertje wat je op gaat maken voor ons. Je kunt ook een gedeelte terugvragen bij CZ, dus dat is ook wel fijn, maar op dat moment kost het wel wat meer geld. |
| ‘I entered the room and he said: “I have not got good news for you”. I then thought: “wow”. It was like a rollercoaster. I had gone alone. I was startled, absolutely. No, I really did not see it coming. It came out of nowhere.’ (male, #1). | Ik kwam binnen en hij zei het al gelijk toen ik binnen was: 'ik heb geen goed nieuws voor je'. Toen was ik echt van: wow. Dan kom je in het circus, eigenlijk een achtbaan. Ik was ook alleen gegaan. Het was wel schrikken, absoluut. |
| ‘Yes, I understand it does influence my prognosis’ (male, #2). | Ja, dat begrijp ik volgens mij dat het mijn prognose beïnvloedt. |
| ‘It was difficult from the moment of the result at the hospital until the scans. Especially after the scans I was wondering: “is it somewhere else, how bad is it?” I thought I was dying.’ (male, #1). | Het was gewoon zwaar van het moment in het ziekenhuis bij de uitslag tot na de scans. Vooral na die scans dat jij je afvraagt: zit het nog ergens anders, hoe ernstig is het. Ik dacht wel dat ik doodging. |
| ‘Everything could have gone faster if people had been more aware that this could be sarcoma’ (male, #1). | Alles had sneller kunnen gaan als mensen zich meer bewust zouden zijn dat dit een sarcoom kan zijn. |
| ‘The key is to start with your general practitioner’ (female, #5). | De kern is toch dat je bij je huisarts begint. |
